# Phytochemicals Characterization and Antidiabetic Efficacy of *Muntingia calabura* L. Leaves Extract: In vitro and in vivo Studies

**DOI:** 10.5812/ijpr-169399

**Published:** 2026-04-11

**Authors:** Raden Maya Febriyanti, Mayra Fauzia, Nurqisthi Iqlimia, Aalbrecht Alby Irawan, Dwintha Lestari, Yoppi Iskandar, Yasmiwar Susilawati, Ajeng Diantini

**Affiliations:** 1 Department of Biological Pharmacy, Faculty of Pharmacy, Universitas Padjadjaran, Indonesia; 2 Herbal Study Center, Universitas Padjadjaran, Bandung, Indonesia; 3 Bachelor Program in Pharmacy, Faculty of Pharmacy, Universitas Padjadjaran, Bandung, Indonesia; 4 Faculty of Mathematics and Natural Science, Universitas Padjadjaran, Bandung, Indonesia; 5 Faculty of Pharmacy, Universitas Muhammadiyah Bandung, Bandung, Indonesia; 6 Department of Pharmacological and Clinical Pharmacy, Faculty of Pharmacy, Universitas Padjadjaran, Bandung, Indonesia

**Keywords:** *Muntingia calabura* L., ABTS Antioxidant Assay, STZ-Induced Diabetic Rats

## Abstract

**Background:**

*Muntingia calabura* L. leaves are traditionally used in West Java to manage hyperglycemia, yet integrated evidence linking their chemical profile with bioactivity remains limited.

**Objectives:**

To profile secondary metabolites in *M. calabura* leaf extract (MCLE), evaluate antioxidat capacity, and perform an assessment of antihyperglycemic efficacy in a diabetic rat model.

**Methods:**

*Muntingia calabura* L. leaves extract was characterized by ultra-high-performance liquid chromatography-high-resolution tandem mass spectrometry (UHPLC–HRMS/MS). Antioxidant activity was measured using the ABTS•⁺ decolorization assay. Antihyperglycemic activity was evaluated in streptozotocin (STZ)-induced diabetic rats treated orally with MCLE (125 or 250 mg/kgBW) for 14 days. Fasting blood glucose (FBG), 2-hour postprandial glucose (2hPP), and body weight were analyzed using two-way repeated-measures analysis of variance (ANOVA).

**Results:**

Ultra-high-performance liquid chromatography-high-resolution tandem mass spectrometry annotated diverse secondary metabolites, with the most intense signals assigned to 5-hydroxy-6,7-dimethoxy-2-phenyl-4H-chromen-4-one (19.99%), isokaempferide (16.94%), and scrophulein (10.90%). *Muntingia calabura* L. leaves extract and its n-hexane and ethyl acetate fractions exhibited ABTS•⁺ radical scavenging activity. In STZ-induced rats, significant group × day interactions were observed for all outcomes (P < 0.001). The 250 mg/kgBW dose attenuated diabetes-associated weight loss and reduced FBG (P < 0.01), and 2hPP at this dose (134.0 ± 4.0 mg/dL) was not significantly different from the normal control or glibenclamide groups (P > 0.05).

**Conclusions:**

*Muntingia calabura* L. leaves extract demonstrated antihyperglycemic activity in STZ-induced diabetic rats, improving both FBG and 2hPP alongside measurable antioxidant capacity.

## 1. Background

Diabetes mellitus is an accelerating global health challenge, with type 2 diabetes mellitus (T2DM) dominating the epidemiology and comprising roughly 96 - 97% of cases worldwide (1, 2). Incidence and prevalence are increasing across most regions. The International Diabetes Federation reported that in 2021, 536.6 million people were affected by diabetes and estimated an increase to 783.2 million by 2045 (2, 3). Crucially, diabetes disproportionately affects low- and middle-income countries, which already host about 81% of cases and are projected to bear most new cases by 2045, with prevalence growth exceeding 100% in lower-middle-income settings compared to 54% in high-income countries (4).

Beyond chronic hyperglycemia, T2DM is characterized by disturbed redox homeostasis; sustained reactive oxygen species overproduction and weakened antioxidant defenses contribute to insulin resistance, β-cell dysfunction, endothelial injury, and the development of microvascular and macrovascular complications (5-7). Current pharmacological treatments do not always target the upstream oxidative and inflammatory pathways. Moreover, their long-term impact can be constrained by adverse effects, financial cost, and limited accessibility in LMIC contexts (8-11). These challenges have renewed interest in ethnobotanical sources as potential drug leads, particularly those with multi-target actions relevant to both glycemic regulation and oxidative stress pathways (12, 13).

*Muntingia calabura* L., locally known as kersen, has been used in traditional medicine in West Java for managing blood glucose levels (12, 14). Past phytochemical studies have shown that *M. calabura* leaves are rich in flavonoids, such as fisetin, pinostrobin, and quercetin, as well as phenolic acids, tannins, and saponins (15, 16). Evidence from in silico and in vitro studies indicates that these compounds possess antioxidant activity and inhibitory effects on carbohydrate-digesting enzymes, indicating potential dual benefits in controlling hyperglycemia and oxidative stress (17, 18).

However, reported enzyme inhibition varies across studies due to variations in extraction methods and experimental conditions (19, 20). In the study of local medicinal plants, *M. calabura* showed strong antioxidant activity but only weak inhibition of carbohydrate-hydrolyzing enzymes (IC_50_ > 500 µg/mL) (21). This finding suggests that bioactivity may involve mechanisms other than direct single-enzyme inhibition, highlighting the need for broader investigative approaches.

## 2. Objectives

This study aimed to characterize the chemical profile and evaluate the antioxidant and antihyperglycemic potential of *M. calabura* leaf extract in the context of T2DM-associated oxidative stress. Specifically, we (1) profiled and putatively annotated the extract’s secondary metabolites using ultra-high-performance liquid chromatography-high-resolution tandem mass spectrometry (UHPLC–HRMS/MS), (2) quantified in vitro antioxidant capacity using the ABTS•⁺ radical scavenging assay, and (3) assessed in vivo antihyperglycemic efficacy in streptozotocin-induced diabetic rats by monitoring fasting blood glucose (FBG) and postprandial (2 h) glucose responses.

## 3. Methods

### 3.1. Plant Material and Ethanolic Extract

Fresh leaves of *M. calabura* L. were collected, authenticated, and processed as described in the previous study (21). To create solvent portions, the primary ethanol-based extract was separated using n-hexane and ethyl acetate. All fractions were concentrated and stored under identical conditions. For subsequent bioassays, samples were freshly reconstituted in methanol for chemical assays or an aqueous vehicle containing 0.5 - 1% DMSO for biological assays.

### 3.2. Proximate Analysis of the Extract

Proximate composition of the dried ethanolic extract was determined at the Central Laboratory, Universitas Padjadjaran. Moisture levels and total ash were measured via gravimetric analysis, while Soxhlet extraction was used for fat content and the Kjeldahl technique for protein. Total carbohydrate content was calculated by difference. All tests were done in triplicate and recorded as % w/w on a dry-weight basis.

### 3.3. Ultra-High-Performance Liquid Chromatography-High-resolution Tandem Mass Spectrometry Metabolite Profiling

Metabolite profiling was performed on a Q Exactive Orbitrap mass spectrometer (Thermo Fisher Scientific) coupled to a UHPLC system through an electrospray ionization (ESI) interface. Chromatographic separation was achieved using an Accucore Phenyl-Hexyl column (100 × 2.1 mm, 2.6 µm) maintained at 40°C. The mobile phase consisted of water and methanol, each supplemented with 0.1% formic acid, delivered at 0.3 mL/min. The extract was prepared at 1 mg/mL. Data were acquired in positive ESI mode using full-scan high-resolution mass spectrometry (HRMS) with data-dependent MS/MS. Metabolites were annotated based on accurate mass, isotopic distribution, and diagnostic fragmentation patterns, supported by matching against mzCloud and ChemSpider reference spectra and cross-checking with previously reported literature.

### 3.4. ABTS•⁺ Radical Scavenging Assay

The antioxidant activity of the crude extract and its n-hexane and ethyl acetate fractions was determined by the ABTS radical cation scavenging method, adapted for use in a 96-well microplate system. The ABTS•⁺ solution was generated by mixing ABTS (7mM) with potassium persulfate (2,45 mM), followed by incubation of the reaction mixture in the absence of light for 12-16 h to allow complete radical formation. For analysis, 20 µL of each sample solution or Trolox reference standard was combined with 180 µL of appropriately diluted ABTS•⁺ working solution. After incubation for 6 minutes, absorbance was read at 734 nm wavelength.

### 3.5. In vivo Antidiabetic Study

All the procedures were conducted in accordance with ethical guidelines approved by the Research Ethics Committee of Universitas Ahmad Dahlan (REC-UAD/02/02/01-2025/006). Male Wistar rats (Rattus norvegiceus), weighing 180 - 220 g, were supplied by the animal research facility at Institut Teknologi Bandung. Prior to experimentation, the animals were housed for a minimum of seven days under standardized laboratory conditions, such as controlled temperature (23 - 25 °C), relative humidity of 50 - 60%, and a 12 h light-dark cycle, with free access to food and drinking water. A randomized, controlled, parallel-group design was used. Following an 8 - 12 h fast, diabetes was induced by a single intraperitoneal injection of STZ at 50 mg/kg in freshly prepared 0.1 M citrate buffer (pH 4.5). Rats received 5% glucose solution for 24 h post-injection to prevent acute hypoglycemia. At 72 h, FBG was measured from the tail vein using a glucometer (Accu-Chek^®^ Instant, Roche), and only rats with FBG ≥ 200 mg/dL were enrolled. Eligible animals were randomized into study groups (n = 6): Normal control (non-diabetic; NaCMC), diabetic control (STZ; NaCMC), MCLE 125 mg/kg, MCLE 250 mg/kg, and glibenclamide 5 mg/kg (positive control). Treatments were administered orally once daily for 14 consecutive days in 0.5% Na-CMC vehicle (1 mL/kg). FBG, 2hPP, and body weight were recorded at day 3 post-STZ as baseline (D1’), and on day 10 (D7’) and day 17 (D14’).

### 3.6. Statistical Analysis

All analyses were run in IBM SPSS Statistics v30. FBG, 2hPP, and body weight are reported as mean ± SD. Treatment effects across the study period were examined using a two-way repeated-measures ANOVA (RM-ANOVA), with group as the between-subject factor and day as the within-subject factor, allowing estimation of the main effects (treatment and time) and the group × day interaction to test whether response trajectories differed among groups. Assumptions of sphericity were checked using Mauchly’s test. When violated, degrees of freedom were adjusted using the Greenhouse-Geisser correction. Pairwise post hoc testing used Bonferroni-adjusted estimated marginal means to emphasize comparisons against diabetic control. Within-group changes were assessed via simple contrasts comparing day 14 to day 1. Statistical significance was defined as P < 0.05, and effect sizes were expressed as partial eta squared (ηp²).

## 4. Results

### 4.1. Proximate Composition of the Ethanolic Leaf Extract

Proximate analysis of the MCLE revealed a lipid-rich matrix. In contrast, ash and crude protein were relatively low (Table 1). 

**Table 1.** Proximate Composition of *Muntingia calabura* L. Leaves Extract

Parameter	Result (% w/w)	Analytical Method
**Moisture**	21.62	Gravimetric
**Total ash**	1.22	Gravimetric
**Crude fat**	51.98	Soxhlet extraction
**Crude protein**	3.03	Kjeldahl
**Carbohydrates (by difference)**	22.15	Calculation

### 4.2. Metabolite Profiling via Ultra-High-Performance Liquid Chromatography-High-Resolution Tandem Mass Spectrometry

**Figure 1.**
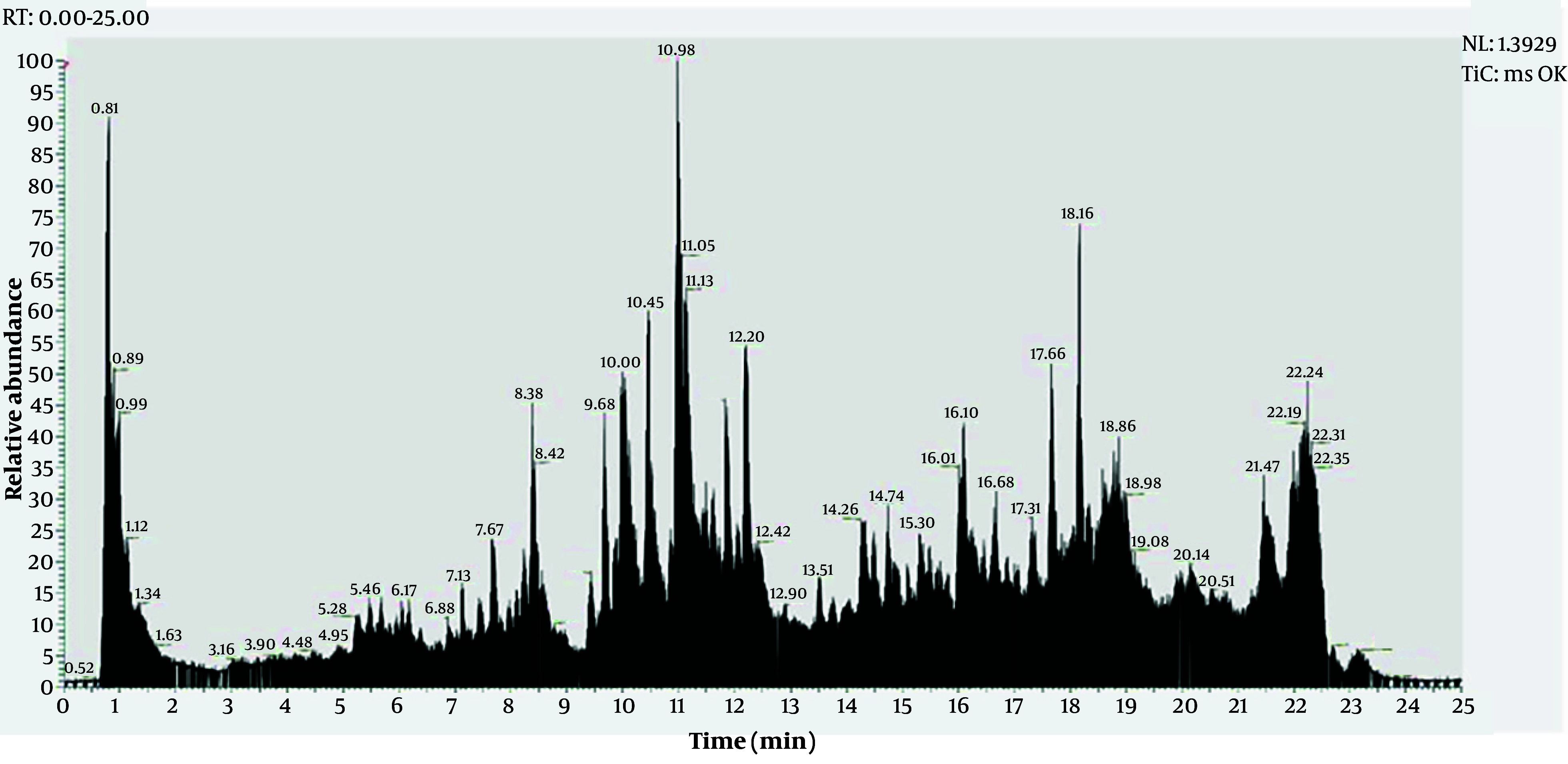
ultra-high-performance liquid chromatography-high-resolution tandem mass spectrometry (UHPLC–HRMS/MS) total ion chromatogram (TIC) of *Muntingia calabura* L. leaves extract

The chemical profile was characterized using UHPLC–Orbitrap HRMS/MS. The total ion chromatogram (TIC) showed a complex metabolite pattern, with most compounds eluting between 0.5 and 23 min (Figure 1). LC–MS/MS characterization reveals a flavonoid-dominant profile, with high signal intensities recorded for multiple flavonols, flavones, and flavanones (Table 2). Within the listed annotated set, the most intense signals were assigned to a polymethoxylated flavone (5-hydroxy-6,7-dimethoxy-2-phenyl-4H-chromen-4-one, 19.99%), the O-methylated flavonol isokaempferide (16.94%), and scrophulein (10.90%), followed by chrysin (6.30%) and the lipophilic antioxidant D-δ-tocopherol (5.15%). This chemical composition is biologically significant, as flavonoids modulate glycemic control through multi-target mechanisms, including the attenuation of oxidative stress and inflammatory cascades that exacerbate insulin resistance, alongside the regulation of AMPK and PI3K/Akt-linked metabolic signaling (22-25).

**Table 2.** LC–MS/MS Metabolites in *Muntingia calabura* L. Leaves Extract

Peak	RT (min)	Putative Compound	Formula	Observed m/z [M+H]+	Class	Relative Abundance (%)
**30**	5.29	Ellagic acid	C₁₄H₆O₈	303.0131	Phenolic acid	0.23
**40**	5.49	Rutin	C₂₇H₃₀O₁₆	611.1601	Flavonol glycoside	0.87
**45**	5.62	Miquelianin	C₂₁H₁₈O₁₃	479.0819	Flavonol glycoside	0.57
**50**	6.16	Kaempferol	C₁₅H₁₀O₆	287.0544	Flavonol	0.62
**36**	6.46	Myricetin	C₁₅H₁₀O₈	319.0446	Flavonol	0.04
**42**	7.44	Quercetin	C₁₅H₁₀O₇	303.0490	Flavonol	0.28
**59**	7.49	Daidzin	C₂₁H₂₀O₉	417.1173	Isoflavone	0.29
**2**	8.11	Apocynin	C₉H₁₀O₃	167.0699	Acetophenone	0.29
**13**	9.66	Pinocembrin	C₁₅H₁₂O₄	257.0803	Flavanone	2.43
**38**	9.68	Diosmetin	C₁₆H₁₂O₆	301.0694	Flavone	1.12
**64**	9.87	Chrysin	C₁₅H₁₀O₄	255.0647	Flavone	6.30
**58**	9.98	Wogonin	C₁₆H₁₂O₅	285.0749	Flavone	3.72
**54**	10.07	Isokaempferide	C₁₆H₁₂O₆	301.0694	Flavonol	16.94
**49**	10.25	Galangin	C₁₅H₁₀O₅	271.0597	Flavonol	0.92
**27**	11.13	Scrophulein	C₁₇H₁₄O₆	315.0853	Flavone	10.90
**39**	11.15	5-hydroxy-6,7-dimethoxy-2-phenyl-4H-chromen-4-one	C₁₇H₁₄O₅	299.0903	Flavone	19.99
**19**	11.75	Glycitein	C₁₆H₁₂O₅	285.0753	Isoflavone	01.02
**1**	14.28	Ursolic acid	C₃₀H₄₈O₃	457.3278	Triterpenoid	0.48
**24**	15.69	Lupeol	C₃₀H₅₀O	427.3.928	Triterpenoid	0.11
**53**	18.16	D-δ-Tocopherol	C₂₇H₄₆O₂	403.3568	Lipid	5.15

The flavonol fraction identified in this analysis, including quercetin (0.28%), kaempferol (0.62%), and myricetin (0.04%), together with their glycosides rutin (0.87%) and miquelianin (0.57%), is particularly relevant given the well-established link between flavonols and both antioxidant and antidiabetic effects. Quercetin has been shown to reduce blood glucose, improve insulin sensitivity, and limit the progression of diabetic complications. These effects are mediated through the AMPK, PI3K/Akt, and Nrf2/ARE pathways. It also influences glucose-handling enzymes and transporters (22-25). Myricetin appears to act in a comparable manner, through the mitigation of oxidative stress and insulin resistance biology (26, 27).

The metabolite profile of MCLE further suggests convergence on pathways governing glycation and oxidative stress. Although ellagic acid represents only 0.23% of the detected constituents, previous studies consistently associate ellagitannin exposure with improvements in FBG, HbA1c, and cardiometabolic risk markers. These outcomes are linked to the suppression of NF-κB, MAPK, and Nrf2/ARE signaling (28-30). Mechanistically, ellagic acid enhances insulin signaling (IRS-1) and glucose translocation (GLUT4) (29, 31). Despite its low relative abundance, these pleiotropic effects position ellagic acid as a high-information marker for bioactivity correlation.

Within the flavone category, chrysin (6.30%) has been reported to have antidiabetic effects, through the regulation of apoptosis- and inflammation-linked mechanisms (32, 33). Wogonin (3.72%) and diosmetin (1.12%) also may contribute to redox modulation. Wogonin activates Nrf2-dependent transcription of phase II antioxidant and cytoprotective genes, including HO-1 and NQO1. Through this pathway, endogenous antioxidant defenses are strengthened and oxidative injury is reduced (34). Additionally, wogonin has been reported to regulate PPAR signaling, which provides a plausible mechanistic bridge between oxidative balance, inflammatory attenuation, and downstream tissue protection (34, 35). These signaling effects are reflected in reproducible reductions in reactive oxygen species (ROS) and inflammatory mediators across experimental contexts (34).

Diosmetin has been shown to act upstream at the Keap1–Nrf2 checkpoint. By alleviating Keap1-mediated repression and enabling Nrf2 nuclear signaling, it enhances downstream antioxidant defenses (36). This upstream effect is associated with measurable reductions in oxidative injury indices, including decreased lipid peroxidation and ROS-associated markers across cardiovascular and tissue models (37, 38). Additionally, rutin (0.87%) adds further mechanistic depth. In STZ-induced diabetic cardiomyopathy models, rutin administration has been shown to reduce hyperglycemia and myocardial injury. It suppresses advanced glycation end-products (AGEs), enhances antioxidant defenses, and increases GLUT4 expression while inhibiting pro-fibrotic mediators such as TGF-β1 and collagen signaling (39).

### 4.3. ABTS Radical Scavenging Activity

The antioxidant capacity of MCLE and its solvent fractions was quantified using the ABTS•⁺ decolorization assay. All samples demonstrated concentration-dependent ABTS•⁺ scavenging, with high inhibition observed across the tested range (15.625 - 250 µg/mL) (Table 3). Notably, the ethyl acetate and n-hexane fractions showed near-complete radical quenching at most concentrations, comparable to the reference antioxidants (Trolox and quercetin), whereas the crude ethanolic extract displayed lower activity at the lowest concentration but approached maximal scavenging at ≥ 62.5 µg/mL. Overall, these data indicate that MCLE contains potent electron/hydrogen-donating constituents, and that antioxidant activity is enriched in the fractionated extracts.

**Table 3.** Radical Scavenging Activity Against ABTS•⁺ Exhibited by *Muntingia calabura* L. Leaves Extractand Derived Fractions ^a^

Concentration (µg/mL)	Trolox	Crude ethanolic extract	Ethyl acetate fraction	n-Hexane fraction	Quercetin
**15.625**	99.873 ± 0.18	68.733 ± 0.45	84.232 ± 0.41	99.232 ± 1.07	89.170 ± 1.62
**31.25**	100.127 ± 0.09	80.317 ± 0.09	99.872 ± 0.18	99.590 ± 0.32	94.547 ± 0.10
**62.50**	100.253 ± 0.24	99.683 ± 1.52	99.590 ± 0.09	99.437 ± 0.39	100.011 ± 4.23
**125.00**	100.937 ± 0.03	100.443 ± 0.72	99.253 ± 0.22	99.462 ± 0.15	100.384 ± 0.11
**250.00**	100.063 ± 0.32	101.647 ± 0.46	99.821 ± 0.32	97.619 ± 0.70	100.153 ± 0.21

^a^ Values are expressed as mean ± SD (n = 3).

### 4.4. In vivo Antihyperglycemic Activity in Streptozotocin-Induced Rats

**Figure 2.**
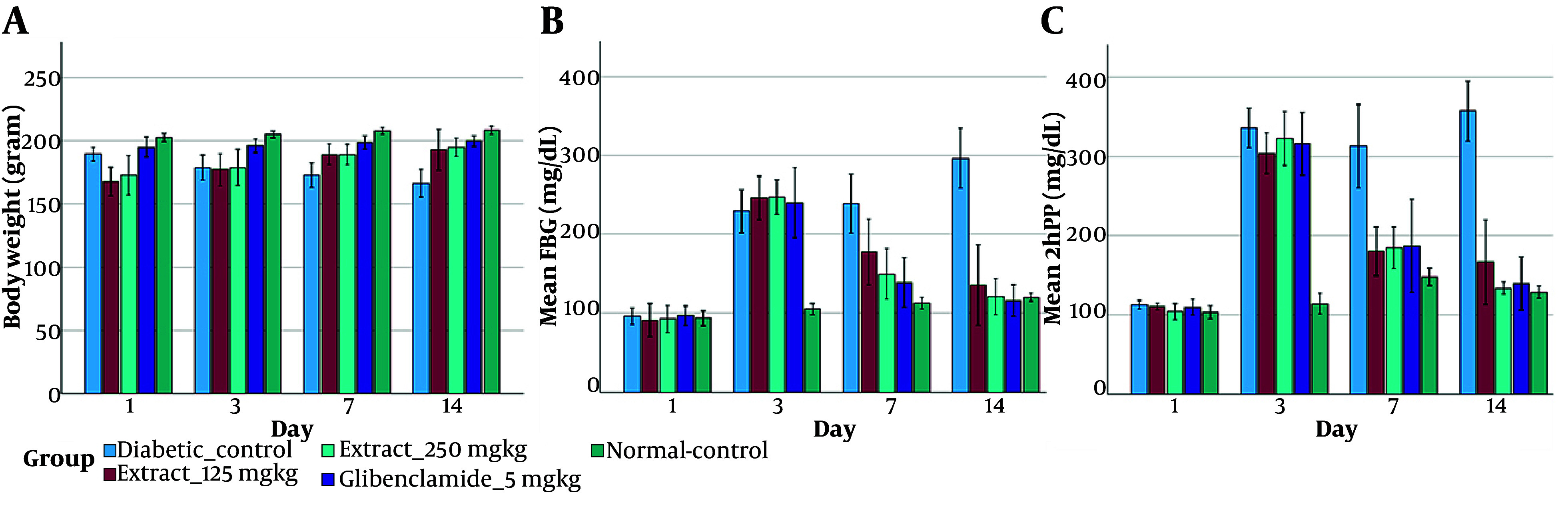
In vivo antihyperglycemic activity in STZ-induced diabetic rats. Data are expressed as mean ± SD (n = 6).

Antidiabetic activity was assessed by monitoring body weight, FBG, and 2 h postprandial glucose throughout the 14-day dosing phase. Body-weight trends are summarized in Table 4 and Figure 2A. Two-way repeated-measures ANOVA indicated a significant group × time interaction [F (6.74, 42.10) = 13.87, P < 0.001, ηp² = 0.69] alongside a significant main effect of treatment group [F (4, 25) = 14.24, P < 0.001]. Diabetic control animals exhibited a progressive decline in body mass (189.5 ± 5.4 g to 166.5 ± 10.9 g), whereas both extract doses (125 and 250 mg/kg) and glibenclamide (5 mg/kg) mitigated diabetes-associated weight loss. Consistent with this pattern, body weights in the treated groups were significantly higher than those in the diabetic control at Day 7 and Day 14 (P < 0.01). Glucose outcomes are presented in Figures 2B - C with numerical values in Table 4. Relative to diabetic control, extract administration at 125 mg/kg and 250 mg/kg produced significant reductions in 2hPP at Day 7 and Day 14 (P < 0.01). Notably, by Day 14, the 250 mg/kg group (134.0 ± 4.0 mg/dL) achieved 2hPP values that were statistically comparable to the normal control (128.7 ± 3.9 mg/dL).

**Table 4.** Effect of Treatments on Fasting Blood Glucose, 2hPP, and Body Weight in Diabetic Rats Over 14 Days ^a^

Variables and Groups	Baseline	Post Induction	Treatment Day 7	Treatment Day 14
**Body weight (g)**				
Normal control	202.67 ± 3.14	205.00 ± 2.76 ^b^	207.67 ± 2.58 ^b^	208.33 ± 3.20 ^b^
Diabetic control	189.50 ± 5.39	178.83 ± 9.81	172.67 ± 9.83	166.50 ± 10.93
Glibenclamide 5 mg/kg	195.17 ± 7.88	196.00 ± 5.44	198.83 ± 5.19 ^b^	199.83 ± 4.17 ^b^
Extract 125 mg/kg	167.67 ± 11.15 ^b^	177.17 ± 12.69	189.17 ± 8.08 ^b^	193.00 ± 16.25 ^b^
Extract 250 mg/kg	172.83 ± 15.55	178.83 ± 14.29	189.17 ± 8.04 ^b^	194.67 ± 7.17 ^b^
**FBG (mg/dL)**				
Normal control	93.83 ± 4.71	105.33 ± 3.56 ^b^	113.00 ± 3.58 ^b^	120.50 ± 2.51 ^b^
Diabetic control	96.33 ± 5.16	229.17 ± 13.57	238.83 ± 18.70	296.50 ± 19.06
Glibenclamide 5 mg/kg	97.00 ± 6.23	240.17 ± 22.27	139.17 ± 15.60 ^b^	116.33 ± 10.01 ^b^
Extract 125 mg/kg	91.50 ± 10.56	246.17 ± 13.78	177.83 ± 20.69 ^b^	136.17 ± 25.47 ^b^
Extract 250 mg/kg	92.83 ± 8.68	247.33 ± 11.02	149.83 ± 15.77 ^b^	121.33 ± 11.41 ^b^
**2hPP (mg/dL)**				
Normal control	103.17 ± 4.22 ^b^	114.17 ± 6.40 ^b^	148.00 ± 5.48 ^b^	128.67 ± 3.88 ^b^
Diabetic control	113.00 ± 2.76	335.83 ± 12.54	312.83 ± 26.38	357.33 ± 18.91
Glibenclamide 5 mg/kg	110.00 ± 4.86	316.00 ± 19.83	186.83 ± 29.25 ^b^	139.33 ± 16.72 ^b^
Extract 125 mg/kg	110.67 ± 2.07	304.00 ± 12.92 ^b^	180.33 ± 15.32 ^b^	166.33 ± 26.58 ^b^
Extract 250 mg/kg	104.17 ± 5.04 ^b^	322.67 ± 17.05	184.50 ± 13.22 ^b^	134.00 ± 3.95 ^b^

^a^ Values are expressed as mean ± SD (n = 6).

^b^ Indicates a statistically significant difference compared to the diabetic control at the same observation point (P < 0.05, Bonferroni-adjusted).

## 5. Discussion

Recent advances in phytochemical and pharmacological evaluation have provided scientific support for the application of *M. calabura* in the management of T2DM. Phytochemical screening of methanolic and ethanolic extracts consistently demonstrates the presence of alkaloids, polyphenols, tannins, and flavonoids, which are commonly associated with metabolic regulation (40, 41). While previous gas chromatography-mass spectrometry (GC-MS) analyses primarily identified triterpenoids such as lupeol and oxadiazole derivatives (42), our UHPLC-HRMS/MS analysis provides a more comprehensive characterization, revealing a chemically complex extract. This is consistent with previous fractionation studies showing that the ethyl acetate fraction contains distinct flavonoid-enriched bands (43). More detailed isolation studies have also identified calaburones with notable α-glucosidase inhibitory activity (17).

In vitro antioxidant findings further support the relevance of *M. calabura* leaves to T2DM pathophysiology, where oxidative stress contributes to β-cell dysfunction and insulin resistance. Prior work showed strong radical-scavenging capacity in methanolic extracts, aligned with high phenolic content (41). In this study, antioxidant capacity was confirmed using the ABTS•⁺ decolorization assay. The observed activity highlights the contribution of flavonoids and phenolic compounds to redox modulation. It also points to a functional overlap between antioxidant activity and the regulation of glucose metabolism (17, 40). Furthermore, a recent study suggests that green-synthesized nanoparticles from leaf extracts can enhance hydrogen peroxide scavenging up to 83.66% (42). This suggests that the intrinsic bioactivity observed in ABTS assays could be further potentiated through nanoformulation.

The in vivo component of this work was designed as a screening-level antihyperglycemic evaluation in STZ-induced diabetic rats, focusing on functional glycemic outcomes rather than mechanistic dissection. Earlier work using alloxan-induced mice demonstrated glucose reduction over a 14-day treatment period (44). In contrast, this study used an STZ-induced model, which is regarded as more reliable and reproducible for sustained hyperglycemia induction (45-47). In this study, administration of the extract at 125 and 250 mg/kg produced significant reductions in both fasting and postprandial blood glucose levels, with the higher dose achieving an antihyperglycemic effect comparable to that observed with glibenclamide. These results demonstrate in vivo glucose modulation under an established hyperglycemic model and provide preliminary pharmacological support for antihyperglycemic activity. However, they should not be interpreted as confirming a specific molecular mode of action or as evidence of clinical efficacy.

Given the heterogeneous metabolite composition and the strong ABTS•⁺ scavenging activity, the observed glycemic improvements are consistent with a multi-constituent, multi-target pharmacology, but any mechanistic interpretation remains hypothesis-based. Although flavonoids such as quercetin and certain flavones have documented α-glucosidase inhibitory and cytoprotective properties in other contexts (17, 48-50), the present study did not directly measure intestinal carbohydrate digestion, insulin secretion, insulin sensitivity, or tissue oxidative stress. Accordingly, we avoid attributing the antihyperglycemic effect to enzyme inhibition, β-cell protection, or antioxidant mechanisms as causal conclusions.

Collectively, our findings provide screening-level evidence that *M. calabura* leaf extract can modulate fasting and postprandial glycemia in an STZ-induced diabetic rat model, supporting further preclinical evaluation without implying clinical efficacy. Although this study did not perform toxicology assessment, previous in vivo oral safety evaluations of *M. calabura* leaf extracts have shown no mortality and no clear treatment-related toxicity following acute high-dose exposure (5000 mg/kgBW). Sub-chronic studies have also not identified significant adverse effects in rats given 50, 250, and 500 mg/kg of *M. calabura* leaf extract orally administered daily for 90 days, providing supportive toxicological context for continued development (51, 52).

However, we also must address several limitations. Because the treatment window was relatively short, long-term efficacy, durability of glycemic control, and dose-response relationships cannot be established. Mechanistic conclusions were based largely on physiological outcomes. Direct measurement of insulin signaling nodes (AMPK/PI3K–Akt axis), inflammatory mediators (NF-κB–linked cytokine networks), and oxidative stress-responsive pathways (Nrf2/ARE target gene induction) was beyond the scope of this work.

### 5.1. Conclusions

This study indicates that *M. calabura* leaves represent a promising phytotherapeutic candidate for T2DM through a coherent multi-target profile linking flavonoid- and phenolic-driven redox modulation with antihyperglycemic activity, consistent with literature on key constituents and diabetes-relevant pathways. Although this study did not include rodent toxicology, previous in vivo oral safety studies of *M. calabura* leaf extracts report no overt toxicity in acute high-dose exposure and no significant adverse findings in repeated-dose designs. Key limitations include the short intervention window and the absence of direct molecular readouts of insulin signaling, inflammatory mediators, and oxidative stress-responsive pathways. Future studies should incorporate chronic diabetic models, targeted molecular mechanism assays, and bioavailability-guided fractionation strategies. Such approaches would help establish exposure–response relationships and advance *M. calabura* toward evidence-based phytopharmaceutical development.

## Data Availability

The dataset presented in this study is available from the corresponding author upon reasonable request during submission or after publication. The data are not publicly available due to institutional data-sharing policies.
